# Supplementary material for: EGFR/SRC/ERK-stabilized YTHDF2 promotes cholesterol dysregulation and invasive growth of glioblastoma
Source: Nat Commun. 2021 Jan 8;12:177. doi: 10.1038/s41467-020-20379-7 (PMC7794382; doi:10.1038/s41467-020-20379-7)
Supplement: Supplementary file 1 — Supplementary Information [file 41467_2020_20379_MOESM1_ESM.pdf]

Supplementary Information

EGFR/SRC/ERK-stabilized YTHDF2 promotes cholesterol dysregulation and invasive growth of glioblastoma

Fang et al.

Supplementary figures

Fang, *et al.* Figure S1

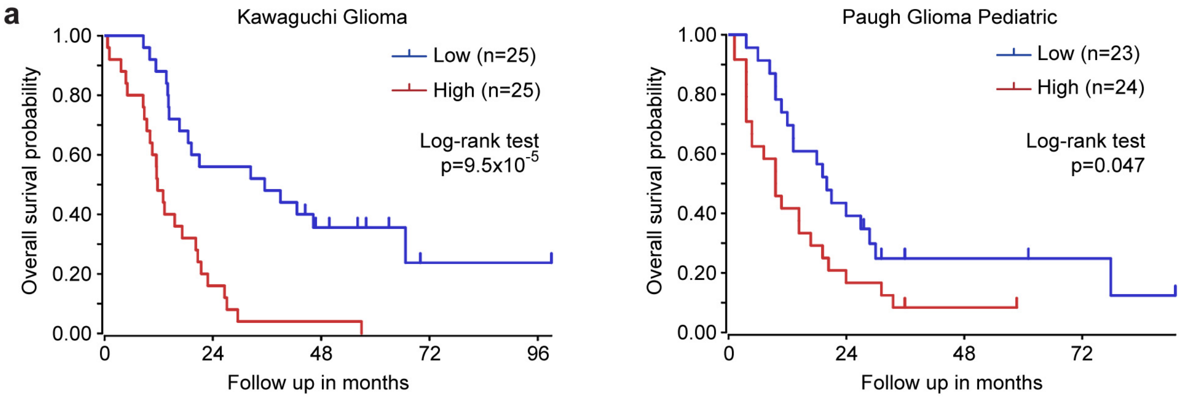

b

Gene	TCGA LGG&GBM n=666	REMBRANDT n=329	French n=273	Kawaguchi n=50	Paugh n=47
	p Value				
YTHDF1	0.1271	0.402	0.639	0.6	0.583
YTHDF2	3.90×10^{-14}	1.00×10^{-6}	3.90×10^{-10}	9.50×10^{-5}	0.047
YTHDF3	0.2736	0.288	0.461	0.446	0.963
YTHDC1	0.0247	1.38×10^{-7}	8.80×10^{-10}	4.80×10^{-4}	0.677
YTHDC2	0.3899	0.299	0.169	0.074	0.33

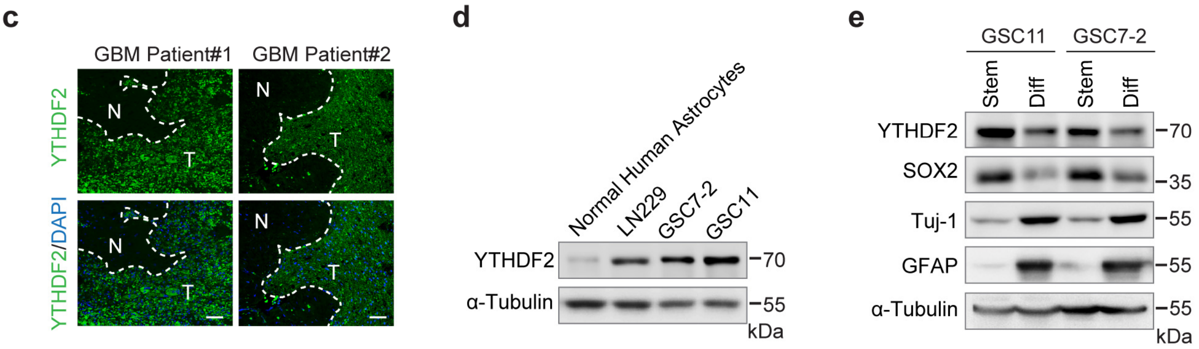

Supplementary Fig. 1 YTHDF2 is highly expressed in glioblastoma and predicts poor prognosis of glioma patients. **a** Kaplan-Meier overall survival plot showing survival rates for glioma patients having *YTHDF2* low expression (blue) and high expression (red) in Kawaguchi and Paugh datasets (two-sided Log-rank test). **b** Summary of YTH m^{6}A readers' prognosis (overall survival) in TCGA, REMBRANDT, French, Kawaguchi, and Paugh datasets. Numbers indicated log-rank p value. Red indicates worse prognosis, green indicates better prognosis, and gray indicates nonsignificant in prognosis (p>0.05). **c** Representative YTHDF2 immunofluorescence staining in GBM specimens. N, non-tumor adjacent cerebrum; T, tumor. Scale bar = 200 μm. Representative image of two independent experiments. **d** Western blotting of

YTHDF2 in normal human astrocytes, GBM cells LN229, GBM-derived stem cells GSC7-2 and GSC11. Representative blot of three independent experiments. **e** Western blotting of YTHDF2, SOX2, Tuj-1 and GFAP in differentiated (diff) GSC11 and GSC7-2 cells. Representative blot of three independent experiments. Source data are provided as a Source Data file.

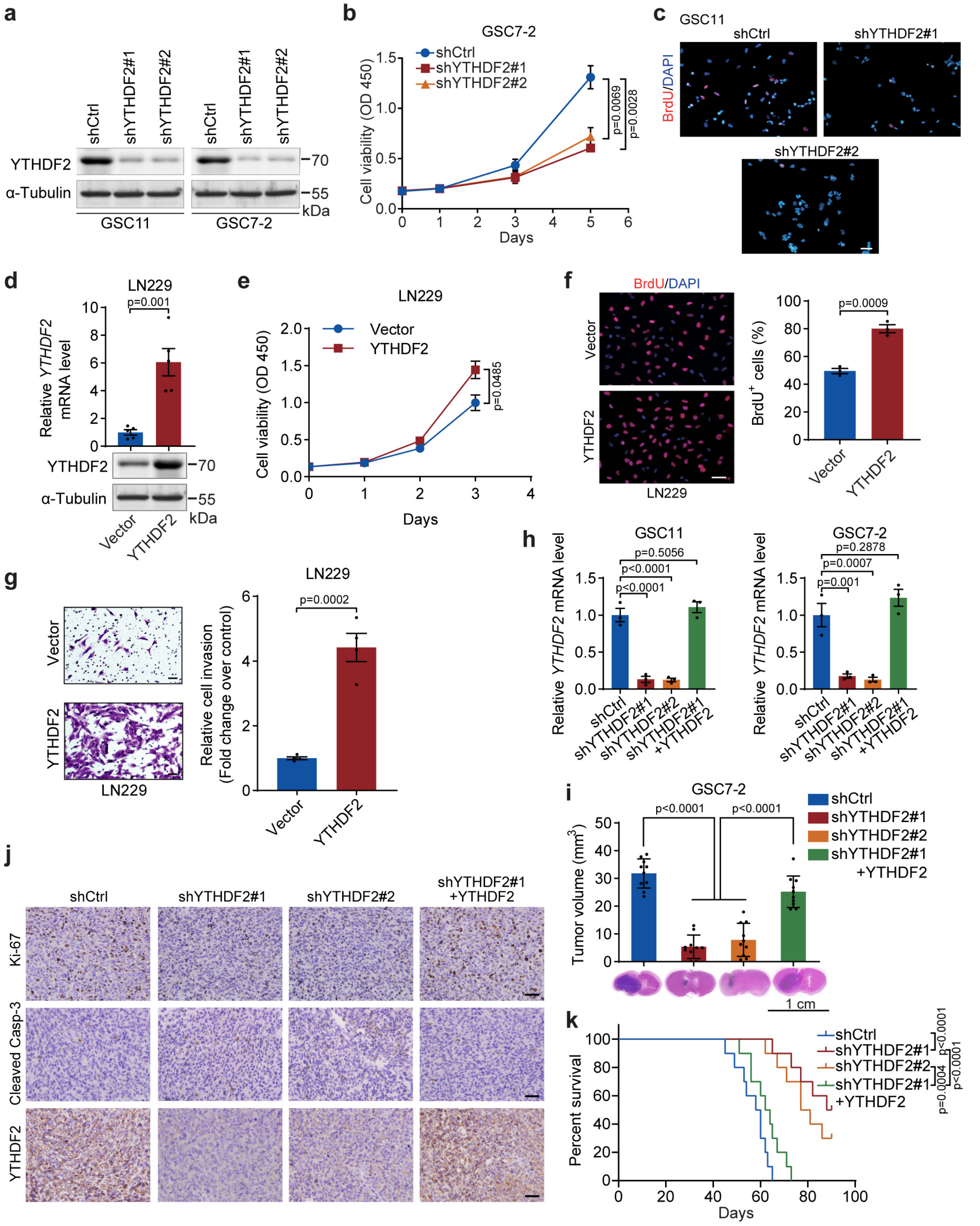
a
shCtrl
shYTHDF2#1
shYTHDF2#2
YTHDF2
α-Tubulin
-70
-55
kDa
GSC11
GSC7-2
b
GSC7-2
shCtrl
shYTHDF2#1
shYTHDF2#2
Cell viability (OD 450)
Days
p=0.0069
p=0.0028
c
GSC11
BrdU/DAPI
d
LN229
Relative YTHDF2 mRNA level
p=0.001
Vector
YTHDF2
e
LN229
Vector
YTHDF2
p=0.0485
f
LN229
BrdU+ cells (%)
p=0.0009
g
Relative cell invasion (Fold change over control)
p=0.0002
h
GSC11
GSC7-2
Relative YTHDF2 mRNA level
p=0.5056
p<0.0001
p<0.0001
p=0.2878
p=0.0007
p=0.001
+YTHDF2
i
Tumor volume (mm3)
p<0.0001
p<0.0001
shYTHDF2#1 +YTHDF2
1 cm
j
Ki-67
Cleaved Casp-3
k
Percent survival
p<0.0001
p<0.0001
p=0.0004

Supplementary Fig. 2 YTHDF2 regulates GBM cell proliferation, invasion and tumorigenesis. **a** Western blotting analysis of YTHDF2 expression in GSC11 or GSC7-2 cells stably expressing shCtrl, shYTHDF2#1 and shYTHDF2#2. Representative blot of three independent experiments. **b** Cell viability of shCtrl and shYTHDF2 GSC7-2 cells was measured by Cell Counting Kit-8 (CCK-8). Data are mean ± S.E.M., n=3 biologically independent experiments (one-way ANOVA Tukey's post-hoc test). **c** Representative immunofluorescence images of BrdU incorporation in GSC11 cells. Scale bar = 100 µm. Representative image of three independent experiments. **d** qPCR and Western blotting analysis of YTHDF2 expression in LN229 cells expressing vector or YTHDF2 plasmid. The quantitative data are presented as mean ± S.E.M., n=5 biologically independent experiments (one-way ANOVA Tukey's post-hoc test). **e** Cell viability of LN229 cells expressing vector or YTHDF2 plasmid was measured by CCK-8. Data are mean ± S.E.M., n=3 biologically independent experiments. (unpaired two-sided t test). **f** Proliferation of the above LN229 cells was assessed by 5-bromo-20-deoxyuridine (BrdU) incorporation for 3 hr. Representative immunofluorescence images of BrdU incorporation in LN229 cells (left panel). Scale bar = 50 µm. BrdU positive cell percentage was quantified (right panel). Data are mean ± S.E.M., n=3 biologically independent experiments (one-way ANOVA Tukey's post-hoc test). **g** *In vitro* invasion assay for the above LN229 cells. Representative invasion images of the cells (left panel) and quantitation of relative cell invasion (right panel). Scale bar=50 µm. Data are mean ± S.E.M., n=4 biologically independent experiments (unpaired two-sided t test). **h** qPCR analysis of *YTHDF2* expression in GSC11 or GSC7-2 cells stably expressing shCtrl, shYTHDF2 and shYTHDF2 plus shRNA-resistant-YTHDF2. The quantitative data are presented as mean ± S.E.M., n=3 biologically independent experiments (one-way ANOVA Dunnett's post-hoc test). **i** Nude mice intracranial tumor assay using shCtrl, shYTHDF2 and shYTHDF2 plus shRNA-resistant-YTHDF2 GSC7-2 cells. Brain sections stained with hematoxylin and eosin (H&E) show representative tumor xenografts. Tumor volumes were calculated using the formula $V = ab^2/2$, where a and b are the tumor's length and width, respectively. Data are mean ± S.D., n=10 mice per group examined over two independent experiments (One-way ANOVA Tukey's post-hoc test). **j** Representative immunohistochemical staining of Ki-67, cleaved Caspase-3 and YTHDF2 in shCtrl, shYTHDF2 and shYTHDF2 plus shRNA-resistant-YTHDF2 GSC11 cells. Scale bar = 200 µm. Representative image of two independent experiments. **k** Kaplan-Meier plots of overall survival of mice injected with the above GSC7-2 cells. n=10 mice per group examined over two independent experiments (two-sided Log-rank test). Source data are provided as a Source Data file.

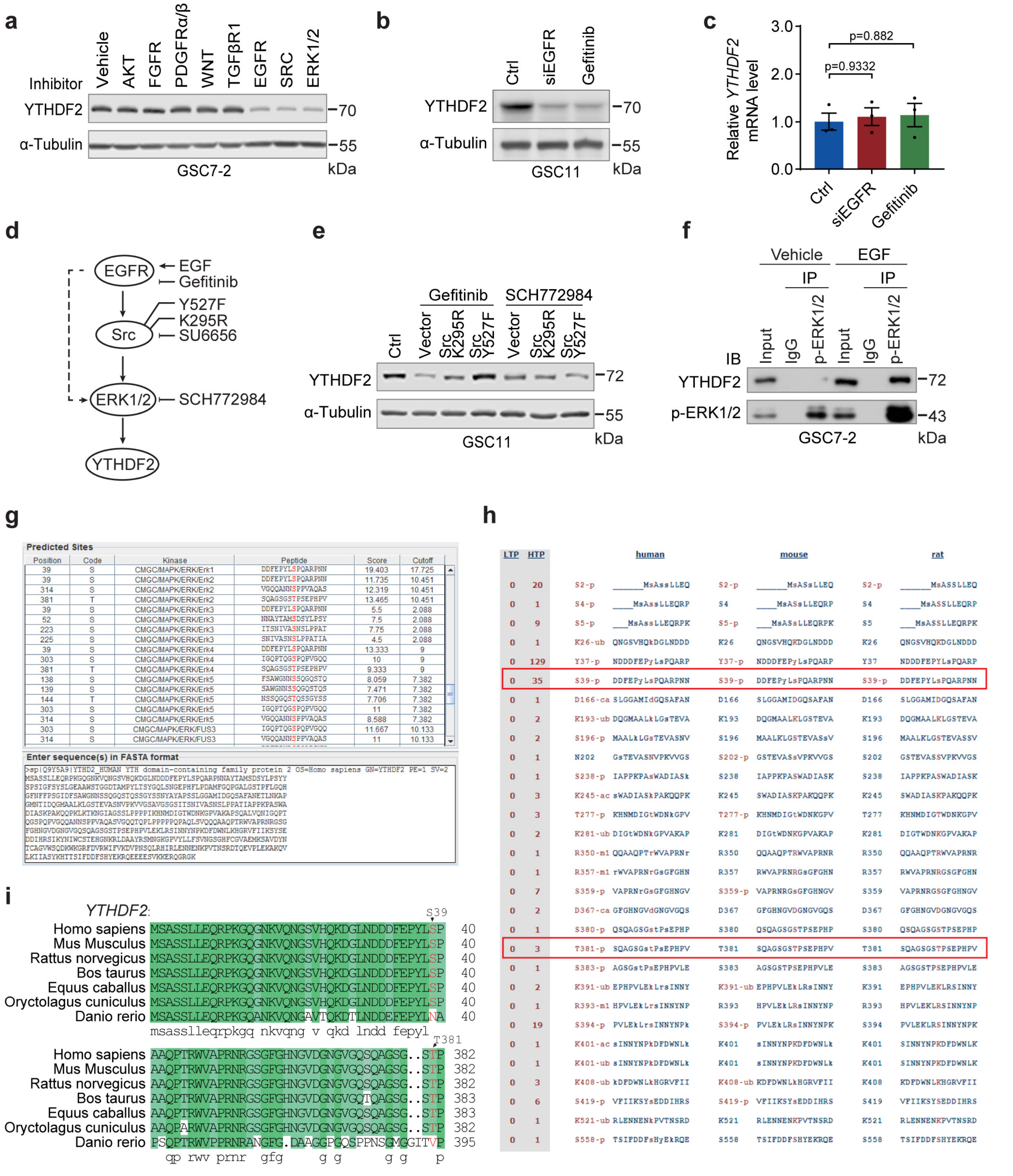
a
Inhibitor
Vehicle
AKT
FGFR
PDGFRα/β
WNT
TGFβR1
EGFR
SRC
ERK1/2
YTHDF2
α-Tubulin
-70
-55
GSC7-2
kDa
b
Ctrl
siEGFR
Gefitinib
YTHDF2
α-Tubulin
-70
-55
GSC11
kDa
c
Relative YTHDF2 mRNA level
p=0.882
p=0.9332
Ctrl
siEGFR
Gefitinib
d
EGFR
EGF
Gefitinib
Src
Y527F
K295R
SU6656
ERK1/2
SCH772984
YTHDF2
e
Gefitinib
SCH772984
Ctrl
Vector
Src K295R
Src Y527F
YTHDF2
α-Tubulin
-72
-55
GSC11
kDa
f
Vehicle
EGF
IP
IB
Input
IgG
p-ERK1/2
YTHDF2
p-ERK1/2
-72
-43
GSC7-2
kDa
g
Predicted Sites
Enter sequence(s) in FASTA format
h
LTP
HTP
human
mouse
rat
i
YTHDF2:
S39
T381
Homo sapiens
Mus Musculus
Rattus norvegicus
Bos taurus
Equus caballus
Oryctolagus cuniculus
Danio rerio

Supplementary Fig. 3 EGFR/SRC/ERK signaling sustains YTHDF2 expression. **a** Western blotting of YTHDF2 in GSC7-2 cells treated with chemical inhibitors of AKT (MK-2206), FGFR (AZD4547), PDGFRα/β (CP673451), WNT (Wnt-C59), TGFβR1 (SB-431542), EGFR (Gefitinib), SRC (SU6656), and ERK1/2 (SCH772984) for 24h. Representative blot of three independent experiments. **b, c** Expression of YTHDF2 protein (b) or mRNA (c) in GSC11 cells interfered with EGFR siRNA or inhibited EGFR with Gefitinib, was measured by Western blotting and qPCR. Representative blot of three independent experiments. The quantitative data are presented as mean ± S.E.M., n=3 biologically independent experiments (one-way ANOVA Tukey's post-hoc test). **d** Schematic description of interventions methods (EGF, Gefitinib, SU6656, SCH772984, K295R and Y527F Src) to dissect the EGFR/SRC/ERK1/2 pathway in YTHDF2 expression. **e** Western blotting of YTHDF2 in GSC11 cells transfected with K295R or Y527F mutated Src with Gefitinib or SCH772984 intervention. Representative blot of three independent experiments. **f** Co-immunoprecipitation (co-IP) of YTHDF2 with phospho-ERK1/2 (Thr202/Tyr204) in whole-cell extracts from GSC7-2 cells cultured with or without EGF. Representative blot of three independent experiments. **g** Predicted phosphorylation sites of YTHDF2 by GPS 3.0 software. **h** Observations of YTHDF2 phosphorylation(s) in PhosphoSitePlus® which extracts high through-put phosphorylation data from published papers (https://www.phosphosite.org/homeAction). **i** Sequence alignment of YTHDF2 from multiple species based on predicted ERK1/2 phosphorylation sites. Identical and conserved residues are highlighted in green and less conserved residues are colored in lighter shades of green. Source data are provided as a Source Data file.

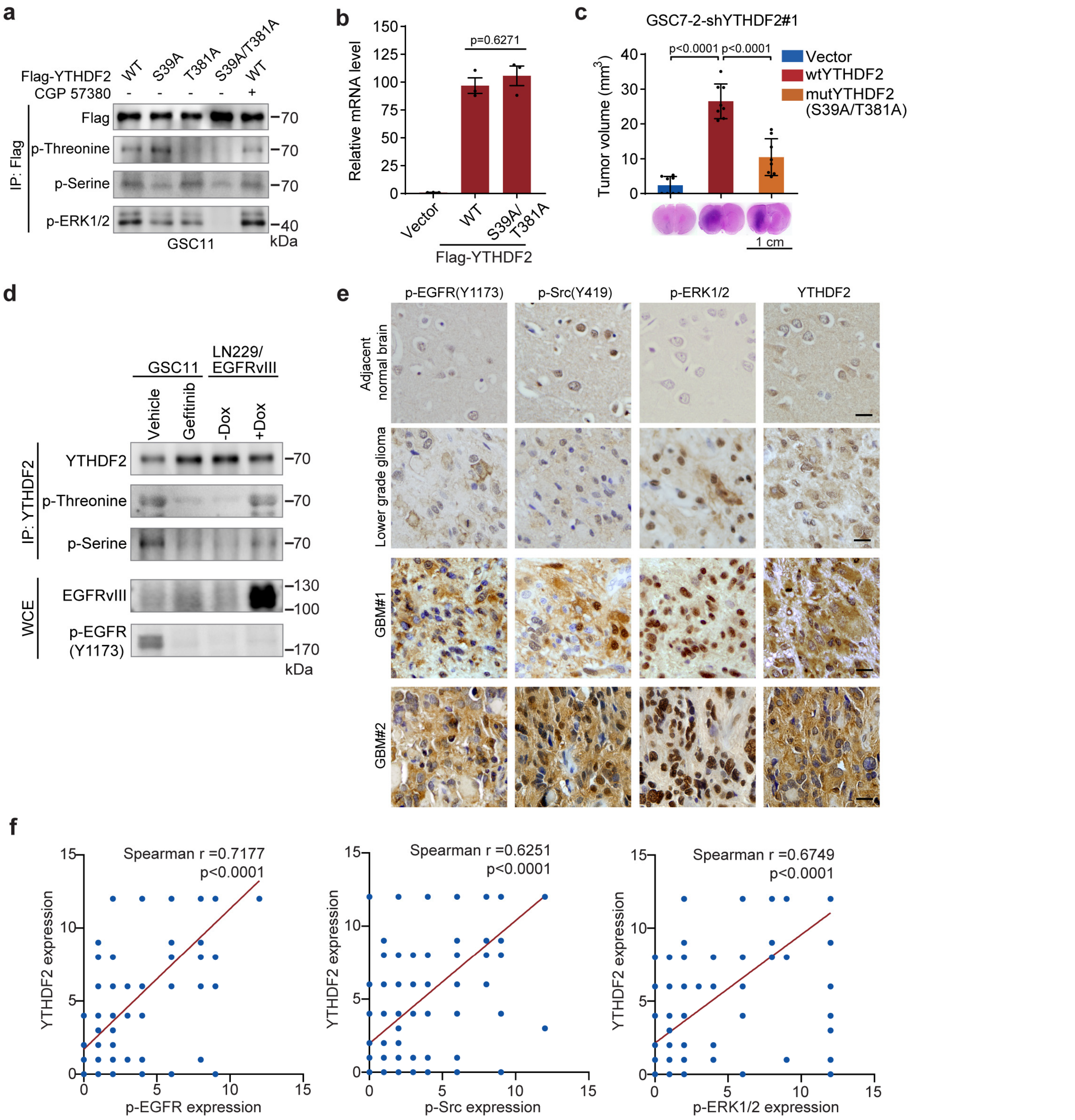

Supplementary Fig. 4 ERK1/2 phosphorylates YTHDF2 at Serine 39 and Threonine 381 to stabilize YTHDF2. **a** Co-IP of phospho-ERK1/2 (Thr202/Tyr204) with WT, S39A, T381A, or S39A/T381A mutated Flag-YTHDF2 in whole-cell extracts from GSC11 cells cultured with

EGF treated with or without 10 μM MNK1 inhibitor CGP 57380. The phosphothreonine and phosphoserine of immunoprecipitated Flag-YTHDF2 were detected using pan-phosphothreonine and pan-phosphoserine antibody respectively. Representative blot of three independent experiments. **b** Flag-YTHDF2 mRNA expression in LN229 cells transfected with Flag-YTHDF2 wild-type or mutant expression constructs. Data are mean ± S.E.M., n=3 biologically independent experiments (one-way ANOVA Tukey's post-hoc test). **c** Nude mice intracranial tumor assay using wild type or S39A/T381A mutated YTHDF2 expressing GSC7-2 cells with YTHDF2 depletion. Brain sections stained with H&E show representative tumor xenografts. Tumor volumes were calculated using the formula $V = ab^2/2$, where a and b are the tumor's length and width, respectively. Data are mean ± S.D., n=8 mice per group examined over two independent experiments (one-way ANOVA Tukey's post-hoc test). **d** Western blotting of phosphothreonine and phosphoserine of immunoprecipitated YTHDF2 in GSC11 cells treated with gefitinib or LN229/EGFRvIII cells treated with Dox. EGFRvIII or phospho-EGFR Y11173 were measured in whole cell extracts (WCE). Representative blot of three independent experiments. **e, f** Immunohistochemical analysis of p-EGFR, p-Src, p-ERK1/2, and YTHDF2 protein expression in human tissue specimens of 14 normal brains, 90 low and lower grade gliomas, and 70 GBMs. Representative IHC image (e) of two independent experiments showed. Tissue was counterstained with hematoxylin. Scale bar, 25 μm. (f) Correlations of the expression of YTHDF2 with the expression of p-EGFR (Y1173), p-SRC (Y419), and p-ERK1/2 in 80 lower grade gliomas and 70 GBM specimens were analyzed by using Spearman correlation test. Source data are provided as a Source Data file.

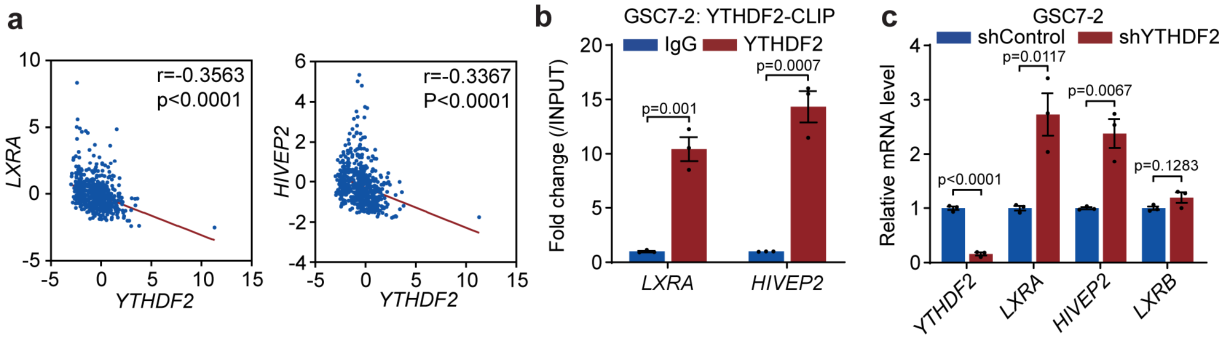

Supplementary Fig. 5 Analyses of potential targets of YTHDF2. **a** The association of the mRNAs of *LXRA* and *HIVEP2* with *YTHDF2* mRNA in lower grade glioma and GBM patients. Data was obtained from the TCGA dataset and analyzed using two-sided Spearman correlation. **b** CLIP-qPCR showing the association of *LXRA* and *HIVEP2* transcripts with YTHDF2 in GSC7-2 cells. Data are mean ± S.E.M., n=3 biologically independent experiments (unpaired two-sided t test). **c** mRNA levels of *YTHDF2*, *LXRA*, *HIVEP2*, and *LXRB* in GSC7-2 cells transfected with control or YTHDF2 shRNA, using qPCR. Data are mean ± S.E.M., n=3 biologically independent experiments (unpaired two-sided t test). Source data are provided as a Source Data file.

Fang, *et al.* Figure S6

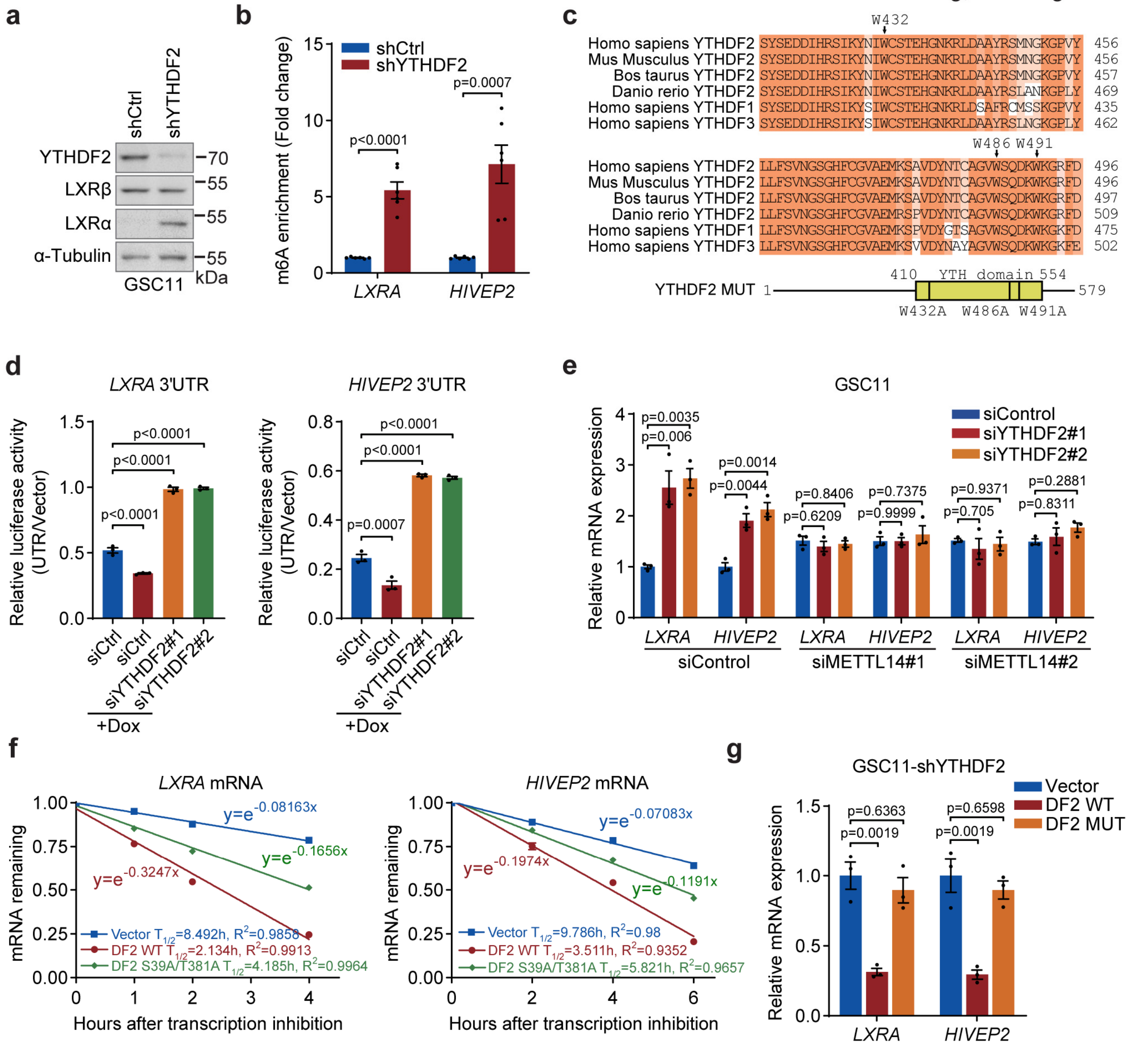

Supplementary Fig. 6 YTHDF2 downregulates *LXRA* and *HIVEP2* through m^{6}A-dependent mRNA decay. **a** Western blotting of LXRα, LXRβ, and YTHDF2 in GSC11 cells stably expressing control or YTHDF2 shRNA. Representative blot of three independent experiments. **b** m^{6}A enrichment of *LXRA* or *HIVEP2* in GSC11 cells stably expressing control or YTHDF2 shRNA. Data are mean ± S.E.M., n=6 biologically independent experiments (unpaired two-sided t test). **c** Sequence alignment of YTHDF2 protein based on selected YTH domains (up panel). Identical and conserved residues are highlighted in orange and less conserved residues are colored in lighter shades of orange. And schematic representation of site mutated in YTHDF2 (YTHDF2 MUT, bottom panel). Arrows indicate residue involved in the recognition of m^{6}A-RNA. **d** Relative luciferase activity of constructs containing 3'UTR of *LXRA* or *HIVEP2*. Data are mean ± S.E.M., n=3

biologically independent experiments (one-way ANOVA Tukey's post-hoc test). **e** mRNA levels of *LXRA* and *HIVEP2* in GSC11 cells co-transfected with control or YTHDF2 siRNAs with METTL14 siRNAs. Data are mean ± S.E.M., n=3 biologically independent experiments (one-way ANOVA Tukey's post-hoc test). **f** Lifetime of *LXRA* and *HIVEP2* mRNAs in YTHDF2-depleted GSC11 cells expressing wild type or S39A/T381A mutated YTHDF2. Transcription was inhibited by actinomycin D. **g** mRNA levels of *LXRA* and *HIVEP2* in YTHDF2-depleted GSC11 cells treated with EGF and transfected with wild type or m^6A recognition defective YTHDF2. Data are mean ± S.E.M., n=3 biologically independent experiments (one-way ANOVA Tukey's post-hoc test). Source data are provided as a Source Data file.

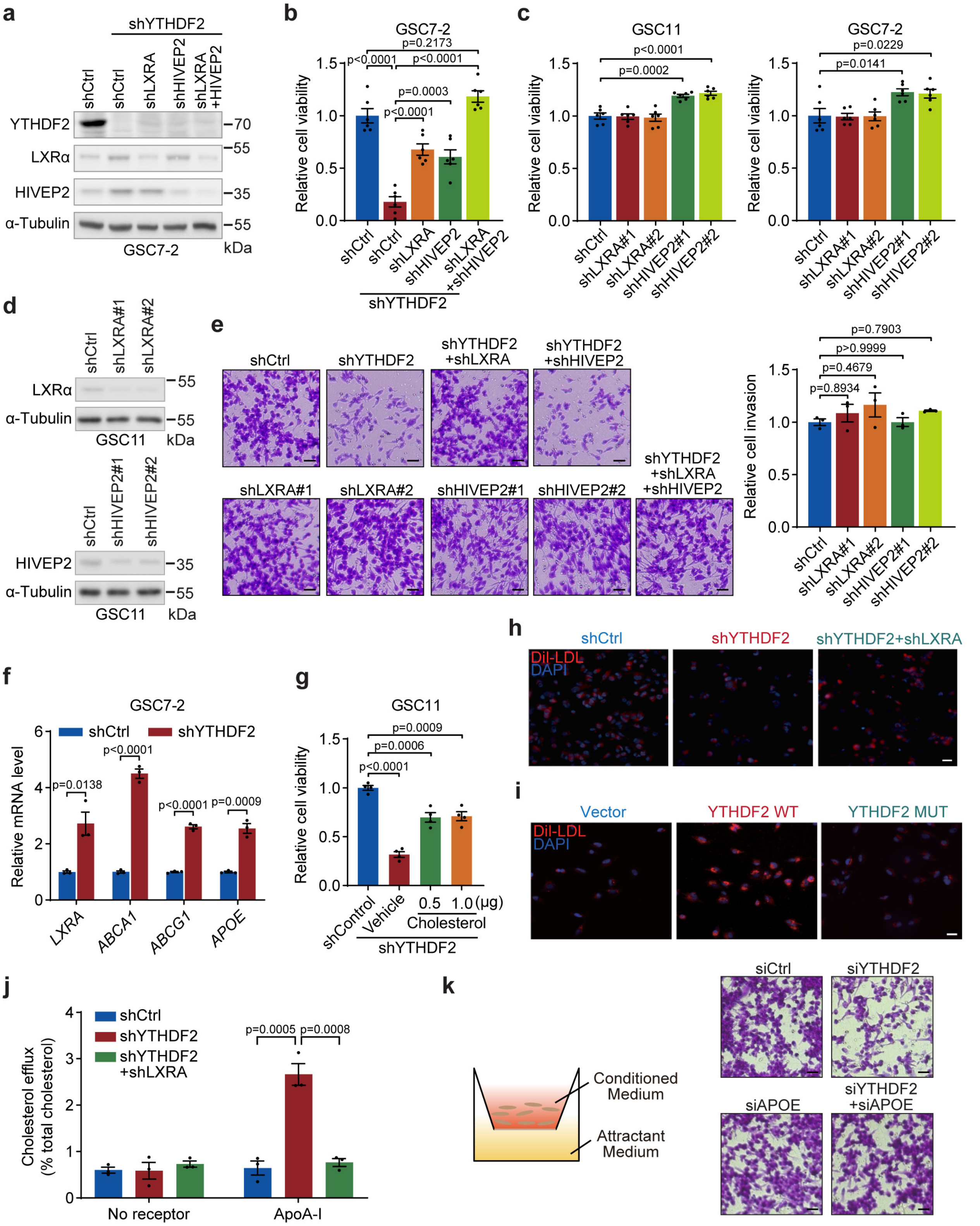
a
shYTHDF2
shCtrl
shCtrl
shLXRA
shHIVEP2
shLXRA +HIVEP2
YTHDF2
LXRα
HIVEP2
α-Tubulin
GSC7-2
kDa
70
55
35
55
b
GSC7-2
Relative cell viability
p=0.2173
p<0.0001
p<0.0001
p=0.0003
p<0.0001
shCtrl
shCtrl
shLXRA
shHIVEP2
shLXRA +shHIVEP2
shYTHDF2
c
GSC11
p<0.0001
p=0.0002
GSC7-2
p=0.0229
p=0.0141
Relative cell viability
shCtrl
shLXRA#1
shLXRA#2
shHIVEP2#1
shHIVEP2#2
d
shCtrl
shLXRA#1
shLXRA#2
LXRα
α-Tubulin
GSC11
kDa
shHIVEP2#1
shHIVEP2#2
HIVEP2
e
shCtrl
shYTHDF2
shYTHDF2 +shLXRA
shYTHDF2 +shHIVEP2
shLXRA#1
shLXRA#2
shHIVEP2#1
shHIVEP2#2
shYTHDF2 +shLXRA +shHIVEP2
Relative cell invasion
p=0.7903
p>0.9999
p=0.4679
p=0.8934
f
GSC7-2
shCtrl
shYTHDF2
Relative mRNA level
p=0.0138
p<0.0001
p<0.0001
p=0.0009
LXRA
ABCA1
ABCG1
APOE
g
GSC11
Relative cell viability
p=0.0009
p=0.0006
p<0.0001
shControl
Vehicle
0.5
1.0 (μg)
Cholesterol
shYTHDF2
h
shCtrl
shYTHDF2
shYTHDF2+shLXRA
Dil-LDL
DAPI
i
Vector
YTHDF2 WT
YTHDF2 MUT
Dil-LDL
DAPI
j
shCtrl
shYTHDF2
shYTHDF2 +shLXRA
Cholesterol efflux (% total cholesterol)
p=0.0005
p=0.0008
No receptor
ApoA-I
k
Conditioned Medium
Attractant Medium
siCtrl
siYTHDF2
siAPOE
siYTHDF2 +siAPOE

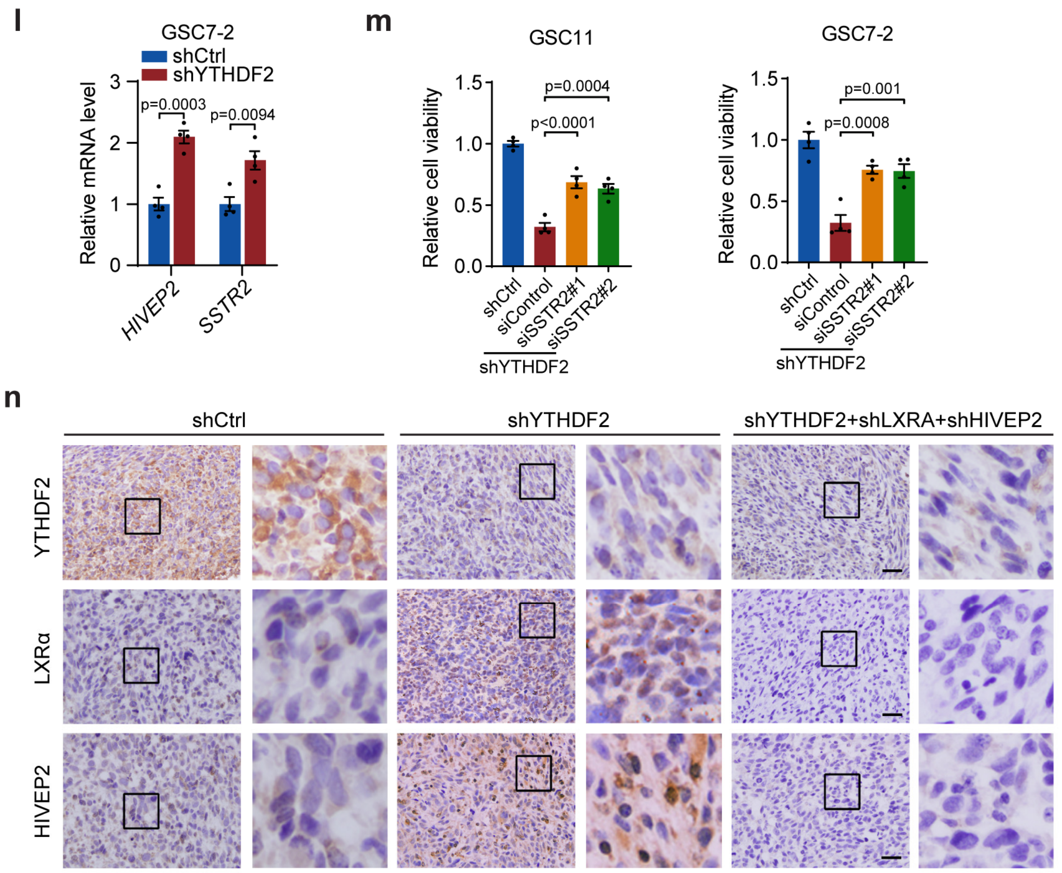

Supplementary Fig. 7 LXRα and HIVEP2 are functionally essential targets of YTHDF2 in cell proliferation, invasion, cholesterol dysregulation and tumorigenesis of GBM cells. **a** Western blotting of YTHDF2, LXRα and HIVEP2 in GSC7-2 cells stably expressing shCtrl, shYTHDF2, or shYTHDF2 plus shLXRA and/or shHIVEP2. Representative blot of three independent experiments. **b** Proliferation of the above GSC7-2 cells was measured by Cell Counting Kit-8 (CCK-8). Data are mean ± S.E.M., n=6 biologically independent experiments (one-way ANOVA Tukey's post-hoc test). **c** Proliferation of GSC11 or GSC7-2 cells stably transfected with LXRA or HIVEP2 shRNAs, was measured by CCK-8. Data are mean ± S.E.M., n=6 biologically independent experiments (one-way ANOVA Tukey's post-hoc test). **d** Western blotting of LXRA or HIVEP2 in GSC11 cells stably transfected with LXRA or HIVEP2 shRNAs. Representative blot of three independent experiments. **e** Representative invasion images (left) and quantification the invasion (right) of GSC11 cells stably expressing shCtrl, shYTHDF2, or shYTHDF2 plus shLXRA or/and shHIVEP2. Representative image of three independent experiments. Scale bar=50 μm. Data are mean ± S.E.M., n=3 wells examined over three independent experiments (one-way ANOVA Tukey's post-hoc test). **f** mRNA levels of *LXRA* and its downstream targets in shCtrl and shYTHDF2 GSC7-2 cells. Data are mean ± S.E.M., n=3 biologically independent experiments (unpaired two-sided t test). **g** Proliferation of GSC11 cells stably transfected with control or YTHDF2 shRNA and treated with

methyl-β-cyclodextrin (MβCD) complexed cholesterol (final concentration of cholesterol is shown). Data are mean ± S.E.M., n=4 biologically independent experiments (one-way ANOVA Tukey's post-hoc test). **h** Representative images of LDL uptake in GSC11 cells expressing shCtrl, shYTHDF2, or shYTHDF2 plus shLXRA. Scale bar = 50 μm. Representative image of three independent experiments. **i** Representative images of LDL uptake in LN229 cells expressing wild-type YTHDF2 (WT) or m^6A recognition defective YTHDF2 (MUT). Scale bar = 50 μm. Representative image of three independent experiments. **j** Cholesterol efflux of GSC11 cells expressing YTHDF2 shRNA with or without LXRA shRNA was determined by scintillation counting. Data are mean ± S.E.M., n=3 biologically independent experiments (one-way ANOVA Tukey's post-hoc test). **k** Schematic description of *in vitro* cell invasion using conditioned culture medium from GSC11 cells transfected with siCtrl, siYTHDF2, siAPOE or siYTHDF2 plus siAPOE (left panel) and representative invasion images of indicated GSC11 cells (right panel). Scale bar=50 μm. Representative image of three independent experiments. **l** Analysis of mRNA levels of *HIVEP2* and its downstream target *SSTR2* in shCtrl and shYTHDF2 GSC7-2 cells. Data are mean ± S.E.M., n=4 biologically independent experiments (unpaired two-sided t test). **m** Proliferation of YTHDF2-depleted GSC11 or GSC7-2 cells transfected with control or SSTR2 siRNAs. Data are mean ± S.E.M., n=4 biologically independent experiments (one-way ANOVA Tukey's post-hoc test). **n** The protein levels of YTHDF2, LXRα and HIVEP2 in brain tumors produced by shCtrl, shYTHDF2 and shYTHDF2 plus shLXRA and shHIVEP2 GSC11 cells were analyzed by IHC. Representative image of two independent experiments. Scale bar = 100 μm. Frames show high-magnification images. Source data are provided as a Source Data file.

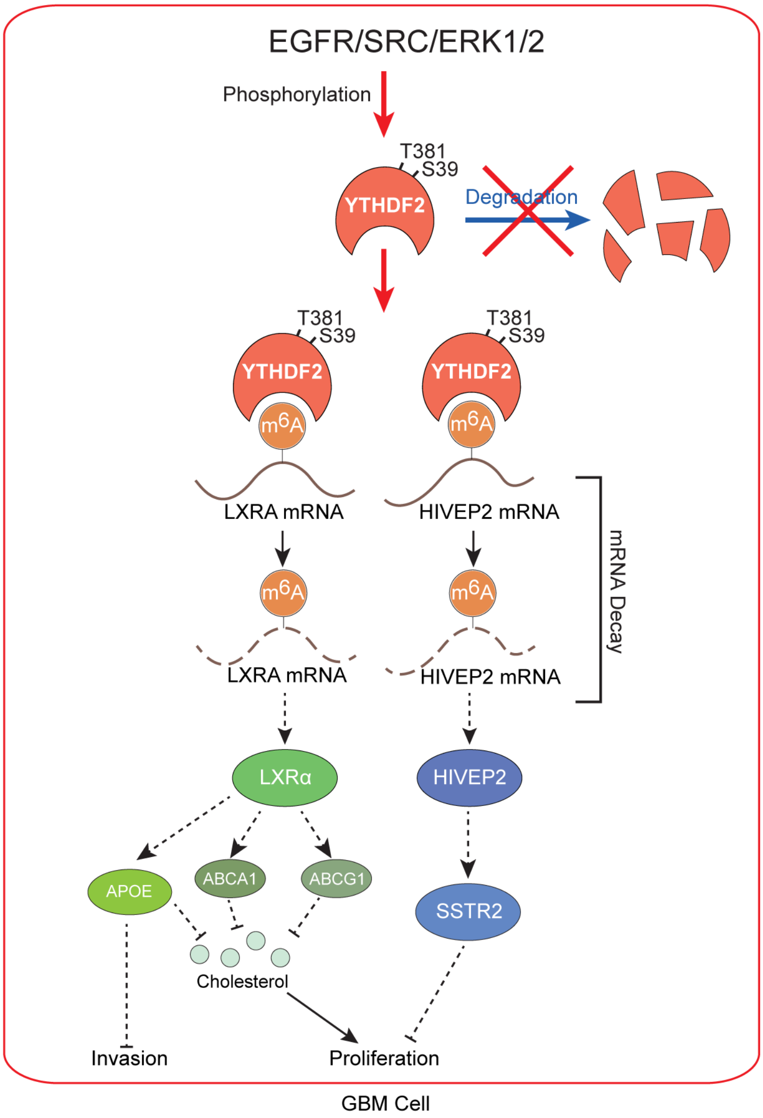

Supplementary Fig. 8 Model for the mechanisms of YTHDF2 overexpression in GBM and YTHDF2's function in GBM tumorigenicity. EGFR/SRC/ERK signaling is highly activated in GBM. ERK1/2 phosphorylates YTHDF2 resulting in the stabilization of YTHDF2 protein. YTHDF2 recognizes the m^{6}A modified mRNAs of target genes, including *LXRA* and *HIVEP2*, and accelerates their degradation. YTHDF2-mediated suppression of LXRα and HIVEP2 expression reduced the expressions of their downstream genes, *APOE*, *ABCA1*, *ABCG1*, and *SSTR2*, which in turn promotes cholesterol dysregulation, cell proliferation, invasion and tumorigenesis of GBM cells.

Supplementary Table 1 Primers used for PCR.

Gene	Sequence
**For qPCR**	
YTHDF2	Forward: 5'-TAGCCAACTGCGACACATTC-3'
	Reverse: 5'-CACGACCTTGACGTTCCTTT-3'
LXRA	Forward: 5'-CATGACCGACTGATGTTC-3'
	Reverse: 5'-CAAACACTTGCTCTGAGTG-3'
LXRB	Forward: 5'-ATCCACTATCGAGATCATGC-3'
	Reverse: 5'-GTCCTTCAAGAAGGTGATAC-3'
HIVEP2	Forward: 5'-AGGGTACAAATCGAATGAAG-3'
	Reverse: 5'-CATACATAAGGCCGAACATC-3'
ABCA1	Forward: 5'-GTGTTTCTGGATGAACCC-3'
	Reverse: 5'-TTCCATTGACCATGATTGC-3'
ABCG1	Forward: 5'-ATCTCCTATGTCAGGTATGG-3'
	Reverse: 5'-AGGGAGATGAAGAAAATCCG-3'
APOE	Forward: 5'-AGGAAGATGAAGGTTCTGTG-3'
	Reverse: 5'-GCAGGTAATCCCAAAAGC-3'
SSTR2	Forward: 5'-AGAAGTCTGAGAAGAAGGTC-3'
	Reverse: 5'-ATATAGGATAGGGTTGGCAC-3'
Flag-YTHDF2	Forward: 5'-GATTACAAGGATGACGACGA-3'
	Reverse: 5'-AGGTTCAAAATCATCATCGTT-3'
GAPDH	Forward: 5'-TGCACCACCAACTGCTTAGC-3'
	Reverse: 5'-GGCATGGACTGTGGTCATGAG-3'
**For PCR**	
3'UTR of LXRA	Forward: 5'-CTAGCTAGCGATGTGCACGAATGACTGTTCTGTC-3'
	Reverse: 5'-CCGCTCGAGACCCTTTGACTCTCTTTTAATGCCA-3'
3'UTR of HIVEP2	Forward: 5'-CTAGCTAGCTCCTTCATCAGAAAAGAGTCAG-3'
	Reverse: 5'-CCGCTCGAGAAGCATGTAATTGTATATTTATTTC-3'

Supplementary Table 2 RNAi oligonucleotides and shRNA sequences.

Name	Sequence
**RNAi oligonucleotides**	
siControl	5'-GAAUACGUACCCCAUUAUA-3'
siYTHDF2#1	5'-UGUAAAACGUGUCGUGAAU-3'
siYTHDF2#2	5'-UUGGCUAUUGGGAACGUCCUU-3'
siEGFR	5'-GAAGGAAACUGAAUUCAAA-3'
siAPOE	5'-CUGCAGCGGGAGACCCUGU-3'
siMETTL14#1	5'-GACUAAGACUUUAGAUCCA-3'
siMETTL14#2	5'-CUACUUGACUGGUUGUACA-3'
siSSTR2#1	5'-UCAUGAGCAUCGACCGAUA-3'
siSSTR2#2	5'-GGUUCAUCAUCUACACUUU-3'
**shRNA**	
shYTHDF2#1	5'-CCGGTACTGATTAAGTCAGGATTAACTCGAGTTAATCCTGACTTAATCAGTATTTTTG-3'
shYTHDF2#2	5'-CCGGGCTACTCTGAGGACGATATTCCTCGAGGAATATCGTCCTCAGAGTAGCTTTTTG-3'
shLXRA#1	5'-CCGGCCGACTGATGTTCCCACGGATCTCGAGATCCGTGGGAACATCAGTCGGTTTTT-3'
shLXRA#2	5'-CCGGAGTTCTCCAGGGCCATGAATGCTCGAGCATTCATGGCCCTGGAGAACTTTTTTTG-3'
shHIVEP2#1	5'-CCGGTTTGGACACTTGGATCTAAATCTCGAGATTTAGATCCAAGTGTCCAAATTTTTG-3'
shHIVEP2#2	5'-CCGGGCCGACCCAATTCATTTGAAACTCGAGTTTCAAATGAATTGGGTCGGCTTTTT-3'
