# Supplementary material for: EGFR/SRC/ERK-stabilized YTHDF2 promotes cholesterol dysregulation and invasive growth of glioblastoma
Source: Nat Commun. 2021 Jan 8;12:177. doi: 10.1038/s41467-020-20379-7 (PMC7794382; doi:10.1038/s41467-020-20379-7)
Supplement: Supplementary file 3 — Description of Additional Supplementary Files [file 41467_2020_20379_MOESM3_ESM.pdf]

Description of Additional Supplementary Files

Supplementary Data 1 Enriched genes by YTHDF2 RNA immunoprecipitation.
Description: Enriched genes with log2FoldChange > 1 and adjust p-value < 0.05 identified by YTHDF2 RNA immunoprecipitation sequencing.

Supplementary Data 2 Overlap genes of MeRIP-seq and RIP-seq.
Description: Overlapping genes identified in both m^{6}A-specific methylated RNA immunoprecipitation sequencing (MeRIP-seq) and YTHDF2 RNA immunoprecipitation sequencing (RIP-seq) in GSC11 cells.

Supplementary Data 3 Differentially expressed genes in siYTHDF2 GSC11 cells.
Description: Differentially expressed genes with fold change >1.5 and adjust p-value <0.05 identified by RNA sequencing in siYTHDF2 GSC11 cells.

Supplementary Data 4 Overlap genes of RNA-seq and RIP-seq.
Description: Overlapping genes between YTHDF2-upregulated genes identified by RNA sequencing and YTHDF2-associated genes identified by MeRIP-seq and YTHDF2 RIP-seq in GSC11 cells.

Supplementary Data 5
cBioPortal_LGG&GBM_PanCancer_dipSamples_SpearmanCorrelation.
Description: Spearman correlation of YTHDF2-targeted genes and YTHDF2 in cBioPortal LGG and GBM PanCancer dipSamples dataset.
