# Supplementary material for: EGFR/SRC/ERK-stabilized YTHDF2 promotes cholesterol dysregulation and invasive growth of glioblastoma
Source: Nat Commun. 2021 Jan 8;12:177. doi: 10.1038/s41467-020-20379-7 (PMC7794382; doi:10.1038/s41467-020-20379-7)
Supplement: Supplementary file 9 — Reporting Summary [file 41467_2020_20379_MOESM9_ESM.pdf]

natureresearch

Corresponding author(s): Chuan He and Suyun Huang

Last updated by author(s): Nov 2, 2020

Reporting Summary

Nature Research wishes to improve the reproducibility of the work that we publish. This form provides structure for consistency and transparency in reporting. For further information on Nature Research policies, see Authors & Referees and the Editorial Policy Checklist .

Statistics

For all statistical analyses, confirm that the following items are present in the figure legend, table legend, main text, or Methods section.

n/a	Confirmed	
☐	☒	The exact sample size (n) for each experimental group/condition, given as a discrete number and unit of measurement
☐	☒	A statement on whether measurements were taken from distinct samples or whether the same sample was measured repeatedly
☐	☒	The statistical test(s) used AND whether they are one- or two-sided<br>*Only common tests should be described solely by name; describe more complex techniques in the Methods section.*
☒	☐	A description of all covariates tested
☐	☒	A description of any assumptions or corrections, such as tests of normality and adjustment for multiple comparisons
☐	☒	A full description of the statistical parameters including central tendency (e.g. means) or other basic estimates (e.g. regression coefficient) AND variation (e.g. standard deviation) or associated estimates of uncertainty (e.g. confidence intervals)
☐	☒	For null hypothesis testing, the test statistic (e.g. F, t, r) with confidence intervals, effect sizes, degrees of freedom and P value noted<br>*Give P values as exact values whenever suitable.*
☒	☐	For Bayesian analysis, information on the choice of priors and Markov chain Monte Carlo settings
☒	☐	For hierarchical and complex designs, identification of the appropriate level for tests and full reporting of outcomes
☐	☒	Estimates of effect sizes (e.g. Cohen's d, Pearson's r), indicating how they were calculated

Our web collection on statistics for biologists contains articles on many of the points above.

Software and code

Policy information about availability of computer code

Data collection	7500 Software v2.0.6 and StepOne™ Software v2.3 were used for collecting Real-time PCR data. Phosphorylation sites of YTHDF2 was predicted by GPS 3.0 software.
Data analysis	GraphPad Prism 8.3 was used for statistical analysis, R-3.6.2 was used for RNA-seq and RIP-seq analysis. Protein quantification in cycloheximide chase analysis and Fluorescence intensity was analyzed using Image J. RNA-sequencing data was mapped using Tophat v2.0.14, and quantified using Cufflink v2.2.1.

For manuscripts utilizing custom algorithms or software that are central to the research but not yet described in published literature, software must be made available to editors/reviewers. We strongly encourage code deposition in a community repository (e.g. GitHub). See the Nature Research guidelines for submitting code & software for further information.

Data

Policy information about availability of data

All manuscripts must include a data availability statement. This statement should provide the following information, where applicable:
- Accession codes, unique identifiers, or web links for publicly available datasets
- A list of figures that have associated raw data
- A description of any restrictions on data availability

The RNA sequencing and RIP sequencing data are deposited at the Gene Expression Omnibus (GEO) repository under the accession number: GSE142828 (https://www.ncbi.nlm.nih.gov/geo/query/acc.cgi?acc=GSE142828). The Cancer Genome Atlas (TCGA) PanCancer Atlas dataset referenced during the study are available in a public repository from the cBioPortal website (https://www.cbioportal.org/), REMBRANDT dataset are available in G-DOC website (https://gdoc.georgetown.edu/gdoc), French, Kawaguchi, and Paugh datasets are available in R2: Genomics Analysis and Visualization Platform (https://hgserver1.amc.nl/cgi-bin/r2/main.cgi). The source data underlying Figs 1f, g, 2a-k, 3a-j, 4a-f, 5g-i, 6a, b, e-n, 7a-o and Supplementary Figs 1d, e, 2a, b, d-i, k, 3a-c, e, f, 4a-d, f, 5a-c, 6a, b, d-g, 7a-g, j, l and m are provided as a Source Data file. All the other data supporting the findings of this study are available from the corresponding author upon reasonable request.

Field-specific reporting

Please select the one below that is the best fit for your research. If you are not sure, read the appropriate sections before making your selection.

- [x] Life sciences
- [ ] Behavioural & social sciences
- [ ] Ecological, evolutionary & environmental sciences

For a reference copy of the document with all sections, see nature.com/documents/nr-reporting-summary-flat.pdf

Life sciences study design

All studies must disclose on these points even when the disclosure is negative.

Sample size
The sample sizes were justified by statistical considerations and statistical power analyses.

Data exclusions
No data were excluded from the analyses.

Replication
For Fig. 2a, b, c, 3d, e, g, j, 5h, 6e, f, h, j, k, 7c, d, e, g, h, i, j, k, and Supplementary Fig. 2b, e, f, h, 3c, 4b, 5b, c, 6d, e, f, g, 7e, f and j, values are calculated from three independent replicated experiments. For Fig. 4c, 5g, 6g, 7l, and Supplementary Fig. 2g, 7g, l and m, values are calculated from four independent replicated experiments. For Fig. 5i, 6l, and Supplementary Fig. 2d, values are calculated from five independent replicated experiments. For Fig. 4d, e, 6m, 6n, 7b, 7f, and Supplementary Fig. 6b, 7b and c, values are calculated from six independent replicated experiments. For Fig. 1e, f, 3j, 2d-k, 4f, 7m-o, and Supplementary Fig. 1c, 2i-k, 4c, e, f and 7n, the results were replicated twice. For representative results in Fig. 1g, 3a, b, c, d, e, f, g ,h, i, j, 4a, b, c, 6a, b, i, 7a and Supplementary Fig. 1d, e, 2a, c, d, f, g, 3a, b, e, f, 4a, d, 6a, 7a, d, e, h, i and k, the results were replicated at least three times.

Randomization
For animal study, mice were randomly divided and grouped. For other experiments, all samples were randomly allocated into experimental groups.

Blinding
The investigators were blinded to group allocation during data collection and data analysis.

Reporting for specific materials, systems and methods

We require information from authors about some types of materials, experimental systems and methods used in many studies. Here, indicate whether each material, system or method listed is relevant to your study. If you are not sure if a list item applies to your research, read the appropriate section before selecting a response.

Materials & experimental systems

n/a	Involved in the study	
[ ]	[x]	Antibodies
[ ]	[x]	Eukaryotic cell lines
[x]	[ ]	Palaeontology
[ ]	[x]	Animals and other organisms
[ ]	[x]	Human research participants
[x]	[ ]	Clinical data

Methods

n/a	Involved in the study	
[x]	[ ]	ChIP-seq
[x]	[ ]	Flow cytometry
[x]	[ ]	MRI-based neuroimaging

Antibodies

Antibodies used
Antibodies used in this study were the following: anti-LXRα (R&D Systems, Cat#PP-K8607-00, 1:500), anti-LXRβ (R&D Systems, Cat#PP-K8917-00, 1:500), anti-HIVEP2 (Invitrogen, Cat#PA5-100756, 1:500), anti-ERK1/2 (137F5) (Cell Signaling Technology, Cat#4695, 1:1000), anti-phospho-ERK1/2 (Thr202/Tyr204) (D13.14.4E) (Cell Signaling Technology, Cat#4370, 1:2000), anti-EGFR (D38B1) (Cell Signaling Technology, Cat#4267, 1:1000), anti-phospho-EGFR (Y1173) (53A5) (Cell Signaling Technology, Cat#4407, 1:1000), anti-Src (Cell Signaling Technology, Cat#2109, 1:1000), anti-phospho-Src (Y419) (Invitrogen, Cat#44-660G, 1:800), anti-α-Tubulin (Santa Cruz Biotechnology, Cat#sc-5286, 1:1000), anti-BrdU (Cell Signaling Technology, Cat#5292, 1:200), anti-N6-methyladenosine (Synaptic Systems, Cat#202003), anti-YTHDF2 (Proteintech, Cat#24744-1-AP, 1:10000), anti-YTHDF2 antibody for RIP (Aviva Systems Biology, ARP67917_P050), anti-FLAG (Sigma-Aldrich, Cat#F1804, 1:1000), anti-SOX2 (Cell Signaling Technology, Cat#3579, 1:1000), anti-GFAP (Cell Signaling Technology, Cat#3670, 1:1000), anti-Tuji-1 (Cell Signaling Technology, Cat#4466, 1:1000), anti-phosphoserine (Millipore, Cat#AB1603, 1:500), anti-phosphothreonine (Cell Signaling Technology, Cat#9386, 1:1000), anti-Ki-67 (Cell Signaling Technology, Cat#9664, 1:400), anti-cleaved caspase-3 (Cell Signaling Technology, Cat#9664, 1:1000), anti-rabbit IgG Alexa Fluor® 488 (Invitrogen, Cat#A-11008, 1:1000), anti-mouse IgG Alexa Fluor® 594 (Invitrogen, Cat#A-11001, 1:1000), anti-rabbit IgG HRP (Abcam, Cat#ab6721, 1:10000), anti-mouse IgG HRP (Santa Cruz, Cat#sc-516102, 1:5000).

Validation
The validation of each primary antibody used in this study for application can be found on the manufacturers' website. For antibody, anti-LXRα (R&D Systems, Cat#PP-K8607-00, 1:500), anti-LXRβ (R&D Systems, Cat#PP-K8917-00, 1:500), anti-HIVEP2 (Invitrogen, Cat#PA5-100756, 1:500), anti-ERK1/2 (137F5) (Cell Signaling Technology, Cat#4695, 1:1000), anti-phospho-ERK1/2 (Thr202/Tyr204) (D13.14.4E) (Cell Signaling Technology, Cat#4370, 1:2000), anti-EGFR (D38B1) (Cell Signaling Technology,

	Cat#4267, 1:1000), anti-phospho-EGFR (Y1173) (53A5) (Cell Signaling Technology, Cat#4407, 1:1000), anti-Src (Cell Signaling Technology, Cat#2109, 1:1000), anti-phospho-Src (Y419) (Invitrogen, Cat#44-660G, 1:800), anti-α-Tubulin (Santa Cruz Biotechnology, Cat#sc-5286, 1:1000), anti-YTHDF2 (Proteintech, Cat#24744-1-AP, 1:10000), anti-FLAG (Sigma-Aldrich, Cat#F1804, 1:1000), anti-SOX2 (Cell Signaling Technology, Cat#3579, 1:1000), anti-GFAP (Cell Signaling Technology, Cat#3670, 1:1000), anti-Tuji-1 (Cell Signaling Technology, Cat#4466, 1:1000), anti-phosphoserine (Millipore, Cat#AB1603, 1:500), anti-phosphothreonine (Cell Signaling Technology, Cat#9386, 1:1000), anti-rabbit IgG HRP (Abcam, Cat#ab6721, 1:10000), anti-mouse IgG HRP (Santa Cruz, Cat# sc-516102, 1:5000), were tested suitable for Western blotting in Human by the manufactures.<br>For antibody, anti-YTHDF2 (Aviva Systems Biology, ARP67917_P050) was also tested for RIP-seq in Zhong Zheng, et al. Cell Rep (2020).<br>For antibody, anti-N6-methyladenosine (Synaptic Systems, Cat#202003) was tested suitable for RNA immunoprecipitation in Human by the manufacture.<br>For antibody, anti-BrdU (Cell Signaling Technology, Cat#5292, 1:200), anti-rabbit IgG Alexa Fluor® 488 (Invitrogen, Cat#A-11008, 1:1000), anti-mouse IgG Alexa Fluor® 594 (Invitrogen, Cat#A-11001, 1:1000), were tested suitable for immunofluorescence by the manufactures.<br>For antibody, anti-Ki-67 (Cell Signaling Technology, Cat#9664, 1:400), anti-cleaved caspase-3 (Cell Signaling Technology, Cat#9664, 1:1000), were tested suitable for immunofluorescence by the manufactures.<br>For Fig. 3h and i, we used IgG as negative control. For YTHDF2, LXRα, HIVEP2, we also used knockdown and/or overexpression for validation. For phospho-EGFR, ERK1/2 and Src, we also used specific inhibitor for validation.

Eukaryotic cell lines

Policy information about cell lines

Cell line source(s)	Human glioma Hs683 and SW1783 cell lines and GBM T98G, U87 MG, LN229 cell line were from the American Type Culture Collection (ATCC). U251 MG cell line were from Sigma (the European Collection of Authenticated Cell Cultures). U87/EGFR cells were a gift from Dr. Frank B. Furnari. GSC11, GSC17, GSC20, GSC23, GSC7-2, and GSC6-27 were obtained from fresh surgical specimens of human primary and recurrent GBMs at MD Anderson Cancer. The characteristics of the GSCs were presented previously (Zhang et al., 2017b). Normal human astrocytes were purchased from LONZA (CAT#CC-2565) and cultured in AGM Astrocyte Growth Medium (CAT# CC-3186, LONZA) following the manufacturer's protocol.
Authentication	Cell lines were authenticated by short tandem repeat (STR) profiling.
Mycoplasma contamination	Cell lines were routinely tested for mycoplasma contamination in every 6 months, and confirmed negative before experimentation.
Commonly misidentified lines (See ICLAC register)	No cell lines used in this study were found in the database of commonly misidentified cell lines that is maintained by The International Cell Line Authentication Committee and NCBI Biosample.

Animals and other organisms

Policy information about studies involving animals; ARRIVE guidelines recommended for reporting animal research

Laboratory animals	Male and female athymic nude mice at 6-8 week age were used in the experiments.
Wild animals	The study did not involve wild animals.
Field-collected samples	No field collected samples were used in the study.
Ethics oversight	All mouse experiments were reviewed and approved by Institutional Animal Care and Use Committees of the University of Texas M D Anderson Cancer Center and the Virginia Commonwealth University.

Note that full information on the approval of the study protocol must also be provided in the manuscript.

Human research participants

Policy information about studies involving human research participants

Population characteristics	Adult patients with glioblastoma.
Recruitment	Enrolled glioblastoma patients were offered the chance for entering the studies under the IRB protocols by research nurses at the UT, M.D. Anderson Cancer Center. Patients participated voluntarily.
Ethics oversight	The use of fresh surgical specimens and archived tissue slides were conducted in accordance with the IRB protocols approved by the Institutional Review Board at the UT, M.D. Anderson Cancer Center with the written informed consents from the patients.

Note that full information on the approval of the study protocol must also be provided in the manuscript.
